# Acupuncture therapy for treating postherpetic neuralgia: A protocol for an overview of systematic reviews and meta-analysis

**DOI:** 10.1097/MD.0000000000023283

**Published:** 2020-11-20

**Authors:** Jie Yu, Mingqi Tu, Yan Shi, Yingjun Liu, Xiaofen He, Fanghui Qiu, Yunyun Xu, Ruohan Sun, Yongliang Jiang, Jianqiao Fang

**Affiliations:** a Department of Acupuncture and Massage, Affiliated Hangzhou First People's Hospital, Zhejiang University School of Medicine; b Department of Neurobiology and Acupuncture Research, the Third Clinical Medical College, Zhejiang Chinese Medical University, Key Laboratory of Acupuncture and Neurology of Zhejiang Province, Hangzhou, China.

**Keywords:** acupuncture therapy, AMSTAR-2, GRADE, overview, postherpetic neuralgia, PRISMA, ROBIS

## Abstract

**Background:**

Postherpetic neuralgia (PHN) is the most common complication and sequela of herpes zoster (HZ) that greatly affects the life and emotional experience of patients. Acupuncture therapy has been confirmed as an effective and safe treatment for PHN. Several systematic reviews (SRs) and meta-analysis (MAs) have reported the evidence of acupuncture therapy for treating PHN. However, the evidence has not been systematically synthesized. This overview aims to synthesize and assess the reliability of evidence generated from these SRs and MAs of acupuncture therapy for PHN.

**Methods:**

We will conduct a systematic search of the China Biology Medicine (CBM), VIP database, Wangfang database, China National Knowledge Infrastructure (CNKI), Pubmed, Cochrane Library, Excerpt Medical Database (Embase), and Web of Science to identify eligible SRs and MAs, from their inception to October 31, 2020. We will use Assessment of Multiple Systematic Reviews-2 (AMSTAR2) for methodological quality assessment, Preferred Reporting Items for Systematic Reviews and Meta-Analyses (PRISMA) for report quality assessment, Grading of Recommendations, Assessment, Development, and Evaluation (GRADE) for the quality of evidence assessment, and ROBIS for the bias assessment. Our reviewers will conduct systematic reviews, qualification evaluation, data extraction, methodological quality, and evidence quality screening in pairs. The outcomes include pain intensity, Quality of life (QoL), Hamilton Anxiety Scale (HAMA), Global impression, and adverse events. All the extracted data will be provided in tabular form to summarize characteristics of each review. The evidence will be a narrative synthesis of the type and content of the intervention and the results reported.

**Results:**

The results of this study will be published in a peer-reviewed journal.

**Conclusions:**

This overview will provide comprehensive evidence of acupuncture therapy for patients with PHN.

**Ethics and dissemination:**

This review will not involve private information of participants, so the ethical approval will not be required. The results will be disseminated in a peer-reviewed journal or conference presentation. Important protocol modifications will be updated on PROSPERO.

**PROSPERO registration number:**

CRD42020178738.

## 1 Introduction

Postherpetic neuralgia (PHN) is the most common complication and sequela of herpes zoster (HZ), which is manifested by neuropathic pain after rash is healed.^[1,2]^ PHN mostly occurs 1 month to 6 months after the rash, and is clinically defined as neuralgia left over 3 months after the HZ rash dissipated.^[3]^ Patients with PHN often suffer from different types of pain, including constant burning pain, paroxysmal tearing pain, hyperalgesia, and allodynia, which may be related to herpes zoster virus nerve infection.^[4]^ Recent studies have shown that 5% to 20% patients with herpes zoster develop with PHN, and the frequency and severity of PHN increase with age.^[1,5,6]^ In China, a higher proportion of patients develop PHN with the ratio of 29.8%, and the rates increased with age.^[7]^ Studies have shown several risk factors for PHN, including prodromal pain, severe acute pain, severe rash, and ophthalmic involvement etc. Among them, old age may be the most obvious risk for PHN.^[8]^ Due to persistent pain, the patients’ quality of life and emotional state are greatly affected. Some patients even experience a permanent loss of independence.^[9,10]^ Western medicine treatments include antidepressants, antiepileptics (gabapentin and pregabalin), opioids (oxycodone, morphine, and methadone), and topical agents, such as lidocaine plasters, topical capsaicin etc.^[11]^ However, these pharmacological therapies are restricted by specific side effects, and the efficacy is limited, only less than 50% patients achieve satisfactory treatment effect (≥ 50% pain relief).^[2]^ Some patients are even treated with nerve blocks.^[12,13]^

Acupuncture therapy, including acupuncture, electroacupuncture, moxibution, cupping, bloodletting etc., has been widely used in different pain conditions. Studies have shown acupuncture has a favorable effect on neuropathic pain.^[14–16]^ Acupuncture therapy has already been confirmed to be effective in treating PHN, not only in relieving pain sensation, but also in improving the quality of life, anxiety, and depression.^[17–20]^

Recently, multiple systematic reviews (SRs)/meta analyses (MAs) have already been conducted to evaluate the effect and safety of acupuncture therapy on PHN.^[21–24]^ High-quality randomized control trials (RCTs) studies can provide strong evidence to guide the treatment of clinical PHN. Overview of SRs and MAs can provide a compile evidence and synthesize the results of multiple SRs and MAs. However, there is still a lack of critically designed overview of SRs and MAs of acupuncture therapy for treating PHN. Therefore, the objectives of this study are as follows: evaluate the methodological quality, report quality and evidence quality and the risk of bias of available SRs and MAs using AMSTAR-2, PRISMA, Grading of Recommendations, Assessment, Development, and Evaluation (GRADE), and ROBIS; summarize the evidence for the effectiveness and safety of acupuncture therapy for treating PHN; provide more reliable, evidence-based medical references for clinical practitioners and researchers.

## 2 Methods

### 2.1 Study registration

This protocol of overview will be performed according to the guideline of the Cochrane Handbook for Systematic Reviews of Interventions. This protocol has already been recorded in the Prospective International Registry of Systematic Review (PROSPERO) (registration number: CRD42020178738, https://www.crd.york.ac.uk/PROSPERO/display_record.php?RecordID=178738). Any changes will be described in our full review.

### 2.2 Inclusion and exclusion criteria

Population, Intervention, Comparison, Outcome and Study (PICOS) strategy will be employed.

#### 2.2.1 Types of studies

SRs and MAs of RCTs which evaluated the efficacy of acupuncture therapy for PHN, published in English and Chinese.

#### 2.2.2 Types of participants

Participants who meet the diagnostic criteria of PHN, that is, pain in a dermatomal distribution sustained for at least 1 month after acute HZ. There are no restrictions on age, sex, or race of participants.

#### 2.2.3 Type of interventions

Acupuncture therapy, including acupuncture, electroacupuncture (EA), moxibustion, warm needling, manual acupuncture, fire needle, bloodletting, cupping, acupoint injection used as intervention for treating PHN, will be included.

#### 2.2.4 Type of comparator (s)/control

The control group's treatment includes conventional drugs, sham acupuncture, placebo, no treatment, and any other therapies other than acupuncture therapy which are considered as comparators in SRs and MAs.

#### 2.2.5 Types of outcome measurements

##### 2.2.5.1 Primary outcomes

Pain intensity, measured by Numerical Rating Scale (NRS), Visual Analogue Scale (VAS), Verbal Rating Scale (VRS), McGill pain score, or other rating scales, will be recommended as the primary outcome.

##### 2.2.5.2 Secondary outcomes

Secondary outcomes mainly include the following aspects:

1. Quality of life (QoL)
2. Hamilton Anxiety Scale (HAMA)
3. Global impression
4. adverse events

#### 2.2.6 Study design

SRs and MAs that contain more than 1 RCT will be included for further study. SRs and MAs without RCTs, reviews, and other overviews will be excluded.

### 2.3 Search methods for identification of studies

We will retrieve 8 electronic databases from their inception to October 31, 2020, which include 4 Chinese databases: China Biology Medicine disc (CBM), VIP database, Wangfang database, China National Knowledge Infrastructure (CNKI), and four English databases: PubMed, Cochrane Library, Excerpt Medical Database (Embase), Web of science. The language will be restricted to Chinese and English. References of the included literatures and PROSPERO database will be also screened to supplement the potential eligible SRs and MAs. The proposed search strategy for PubMed is presented in Table 1.

**Table 1:** Search strategy in PubMed.

Order	Search items
#1	MeSH: “postherpetic neuralgia”
#2	Ti/Ab: “postherpetic neuralgia” OR “postherpesneural gia” OR “postherpetic neuringgia” OR “PHN” OR “Herpes zoster” OR “Shingles”
#3	#1 OR #2
#4	MeSH: “acupuncture therapy” OR “acupuncture” OR “moxibustion” OR “cupping” OR “bloodletting” OR “needle” OR “acupoint”
#5	Ti/Ab: “acupuncture therapy” OR “acupuncture” OR “moxibustion” OR “cupping” OR “bloodletting” OR “needle” OR “acupoint” OR “electroacupuncture” OR “needling” OR “acupoint injection” OR “warm needling”
#6	#4 OR #5
#7	MeSH: “Systematic Review” OR “Meta-Analysis” OR “Systematic Reviews as Topic” OR“Meta-Analysis as Topic”
#8	Ti/Ab: “Systematic Review” OR “Meta-Analysis” OR “Network Meta-Analysis”
#9	#7 OR #8
#10	#3 AND #6 AND #9

### 2.4 Studies selection

**Figure 1:**
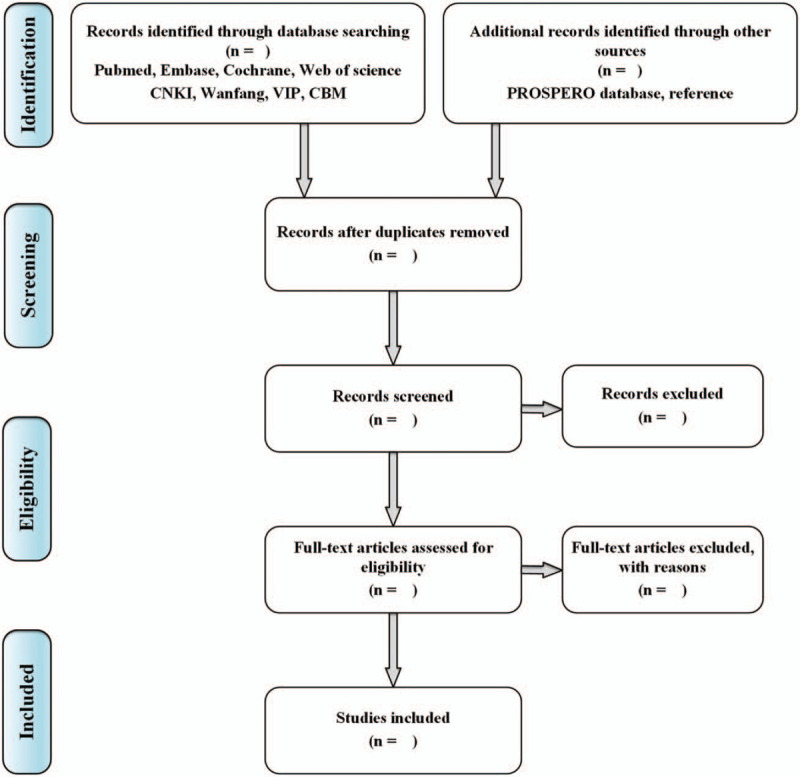
Flowchart of literature selection.

Two independent reviewers (JY and MT) will screen the included databases for related studies. The eligible articles will be imported into Noteexpress 3.2.0 and duplicate articles will be identified and deleted. Then 2 reviewers will review full text independently to determine the inclusion of SRs and MAs. If the study has incomplete information, JY will try to contact the corresponding author of the study for full research details. Any discrepancies will be solved by introducing a third researcher (JF) for judgment. Study selection will be performed in accordance with the PRISMA flowchart (Fig. 1).

### 2.5 Data extraction

Two reviewers (JY and MT) will independently extract the following information from each included study: first author, year, country, number of RCTs enrolled, quality assessment tool for RCTs included in SRs and MAs, interventions, comparisons, including style of acupuncture therapy, outcome measures (primary and secondary outcomes), data synthesis methods, main results, and conclusions. Any disagreement will also be solved by introducing a third researcher (JF) for judgment.

### 2.6 Evaluate the methodological quality of included studies

Two reviewers (JY and YL) will evaluate the methodological quality of included studies independently, using AMSTAR-2, which is an update of AMSTAR.^[25]^ Assessment of Multiple Systematic Reviews-2 (AMSTAR-2) is a critical appraisal tool for SRs that include randomized or nonrandomized studies and become used commonly to assess the quality of SRs and MAs included in overviews.^[26]^ AMSTAR-2 includes 16 items, with each of the 16 criteria given a rating of “yes” (definitely done), “no” (definitely not done), “can’t report” (unclear if completed), or “not applicable” based on information provided by the SRs. Reviewers will evaluate the evaluation when the criterion is met.

### 2.7 Evaluation of the reporting quality of the included studies

Additionally, PRISMA will be applied to assess report quality of SRs and MAs. Two authors (MT and YL) will evaluate the reports’ quality of each study using PRISMA. PRISMA is a 27-item list. Each checklist item will be evaluated as yes, no, or partially Yes to indicate compliance.^[27]^

### 2.8 Evaluation of the evidence quality of the included studies

The evaluation of the evidence quality of the included studies will be conducted by 2 reviewers (JY and YL), using the GRADE approach. GRADE specifies 4 categories: high, moderate, low, and very low.^[28]^ Two reviewers will evaluate the evidence quality of the outcomes of the included SRs and MAs independently, and describe the downgraded or upgraded factors that may affect the evidence quality to guarantee the reliability and transparency of results. If there are any disagreements, they will be solved by introducing a third researcher (JF) for judgment.

### 2.9 Evaluation of the risk of bias of the included studies

Two authors of this review (MT and YS) will assess the risk of bias of the included studies, using ROBIS tool.^[29]^ The ROBIS is a tool to assess the risk of bias of SRs, which involves assessment of 4 domains: study eligibility criteria; identification and selection of studies; data collection and study appraisal; and synthesis and findings. The evaluation of the risk of bias is associated with each domain which will be judged as “low risk,” “high risk,” or “unclear risk.”

### 2.10 Dealing with lost data

If no specific or insufficient data exists in the included SRs and MAs, the author will contact the original author of the article by email or telephone to get the necessary information. Insufficient data will be discarded if we fail to obtain enough data. The analysis will be conducted based on available data, and the potential impact of missing data will be discussed.

### 2.11 Synthesis of data

This overview will analyze SRs and MAs for PHN. General characteristics of the included studies include the total sample size of SRs and MAs, interventions, and their effect size and related 95% CIs. AMSTAR2 will be used for the SRs and MRs methodological quality assessment, PRISMA will be applied to assess report quality, and GRADE for the quality of evidence and ROBIS for the bias, which will be conducted in tabular form for each review. Additionally, the total percentage and the 95% CI of each item will be calculated. The quality of evidence will be detailed in the form of tables. We will combine the reviews in a narrative summary, structured around the type and content of interventions and the reported results. Besides, we will use κ value^[30]^ to measure the agreement degree between the 2 reviewers: κ < 0.4 for poor agreement, 0.4 to 0.75 for fair agreement, κ > 0.75 for excellent agreement.

In addition, the acupuncture therapy treatments will be assessed at SRs and MAs level. We will extract pooled relative risk (RR) or pooled odds ratio (OR) for dichotomous outcomes, and pooled weighted mean difference or standardized mean difference for continuous outcomes which will be also reported with 95% confidence interval (CI). The I^2^ values will be described for reporting heterogeneity across RCTs, with 0% to 25% representing low heterogeneity, 26% to 50% representing medium heterogeneity, and above 50% representing high heterogeneity.^[31]^

## 3 Discussion

PHN belongs to a common kind of neuropathic pain with unsatisfactory clinical efficacy. Many patients still suffer from pain after taking the medication, which greatly affects the quality of life. Acupuncture therapy has been shown to be effective in treating pain caused by PHN and can also improve mood disorders such as anxiety and depression. And acupuncture therapy has been confirmed to be safe without side effects.^[23]^ Previous study has found that the mechanism of acupuncture therapy for PHN may be related to protomics indexes in serum, such as Lymphotoxin beta/TNFSF3, IL-19, Neuritin, NCAM-1/CD56, and PECAM-1/CD31.^[32]^ Peripheral and serum substance P (SP) and TRPV1 neuronal activation will be also associated with the curative effect of acupuncture therapy.^[17,33]^ Many SRs and MAs have evaluated the efficacy and safety of acupuncture therapy for PHN, and have evaluated the quality of RCT. But an overview for SRs and MAs is still lacking in this field. This study will be an overview of SRs and MAs of acupuncture therapy for treating PHN. We plan to find out an optimized acupuncture therapy method for treating PHN, and improve the efficacy and safety of PHN with acupuncture therapy. The results of this review will ascertain the credibility of current evidence. Furthermore, it can provide future research direction about PHN and PHN treatments.

However, there are several limitations of this study: Language limitations may leave out studies in other languages. Different operation methods of acupuncture therapy should also be taken into consideration. These may cause deviations of results and ultimately affect the reliability of this study.

## Author contributions

**Conceptualization:** Jie Yu, Mingqi Tu.

**Data curation:** Jie Yu, Mingqi Tu, Yan Shi, Yingjun Liu.

**Formal analysis:** Yan Shi, Xiaofen He, Fanghui Qiu.

**Investigation:** Jie Yu, Xiaofen He, Yongliang Jiang.

**Methodology:** Jie Yu, Mingqi Tu, Yingjun Liu.

**Software:** Yunyun Xu, Ruohan Sun.

**Supervision:** Yongliang Jiang, Jianqiao Fang.

**Writing – original draft:** Jie Yu, Mingqi Tu, Yan Shi.

**Writing – review & editing:** Xiaofen He, Fanghui Qiu, Yongliang Jiang, Jianqiao Fang.
